# Rare Case of Open Comminuted Scaphoid Fracture Dislocation: Treatment With Free Bone Graft and Locking Plate

**DOI:** 10.7759/cureus.61055

**Published:** 2024-05-25

**Authors:** Makoto Fukuda, Masahiro Miyashita, Masataka Yasuda

**Affiliations:** 1 Department of Orthopaedic Surgery, Baba Memorial Hospital, Sakai, JPN; 2 Department of Traumatology and Critical Care Medicine, Osaka Metropolitan University Graduate School of Medicine, Osaka, JPN

**Keywords:** locking plate, free bone graft, open scaphoid fracture, scaphoid fracture dislocation, comminuted scaphoid fracture

## Abstract

To the best of our knowledge, there are no reports in the literature of an open comminuted scaphoid fracture dislocation. We present such a rare case. The case report illustrates the case of a 58-year-old right-handed press operator who injured his left wrist when his hand got caught in a press machine. He received initial treatment at another hospital and was subsequently referred to our hospital. Eight days after the injury, surgery was performed under the brachial plexus block. Successful bone fusion was achieved through volar locking plate fixation, primary free bone grafting from the radius, and Kirschner wire fixation. Our case report may be a valuable resource for the treatment of similar injuries.

## Introduction

Scaphoid fractures are relatively common, while comminuted and segmental fractures are less commonly reported [1-3]. Furthermore, to the best of our knowledge, there are no reports in the literature of an open comminuted scaphoid fracture dislocation. Such injuries are at risk for nonunion due to a variety of factors, including, interfragmentary instability, retrograde vascular supply, and lack of soft tissue attachments on a largely cartilaginous surface [4]. Several options exist for treating scaphoid fractures and nonunions. Treatment of scaphoid nonunion with plates has been described previously for difficult cases that were not ideally suited to compression screw fixation [5]. We experienced a rare case of an open comminuted scaphoid fracture dislocation. We report such a difficult case treated with a locking plate and free bone grafting from the radius.

This article was previously presented at the 47th Annual Meeting of the Japanese Society for Fracture Repair from July 2, 2021, to July 3, 2021.

## Case presentation

**Figure 1:**
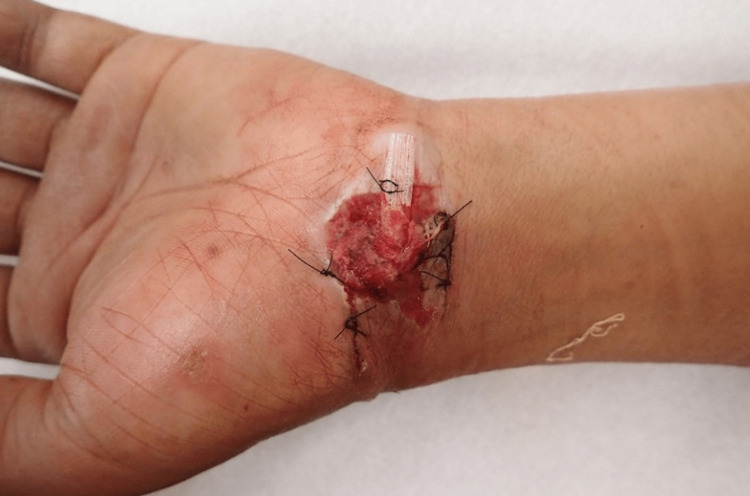
Photograph of the left wrist at the time of the first visit to our hospital. An open wound was found on the volar side of the left wrist.

A 58-year-old right-handed press operator injured his left wrist when his hand got caught in a press machine. He received initial treatment at another hospital and was subsequently referred to our hospital. An open wound was observed on the volar side of his wrist (Figure 1).

**Figure 2:**
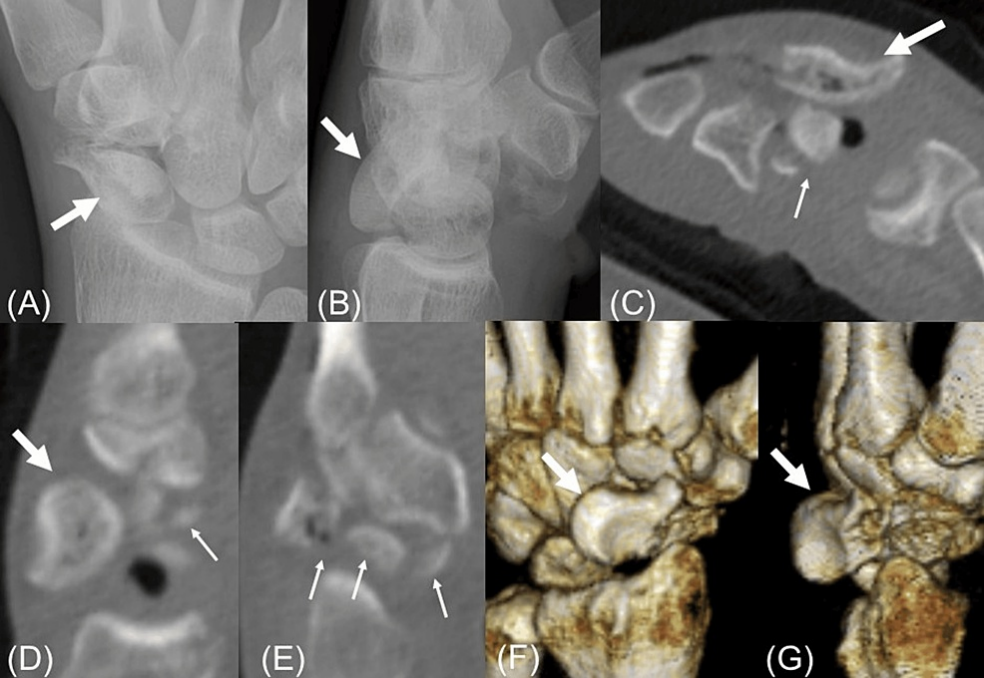
Initial radiographs and computed tomography scans. Initial radiographs and computed tomography (CT) scans show the scaphoid fracture and dorsal dislocation. The thick arrows show the proximal fragment of the scaphoid. The thin arrows show the distal fragment of the scaphoid. (A) Antero-posterior film of the left wrist shows a comminuted scaphoid fracture. (B) The lateral film of the left wrist shows dorsal dislocation of the proximal fragment of the scaphoid. (C-E) Axial and sagittal CT images show a comminuted scaphoid fracture with dorsal dislocation of the proximal fragment. (F, G) 3D CT reconstruction of the wrist in different views shows dorsal dislocation of the proximal fragment of the scaphoid.

Radiographs and computed tomography (CT) scans showed a comminuted scaphoid fracture with dorsal dislocation of the proximal fragment (Figure 2).

**Figure 3:**
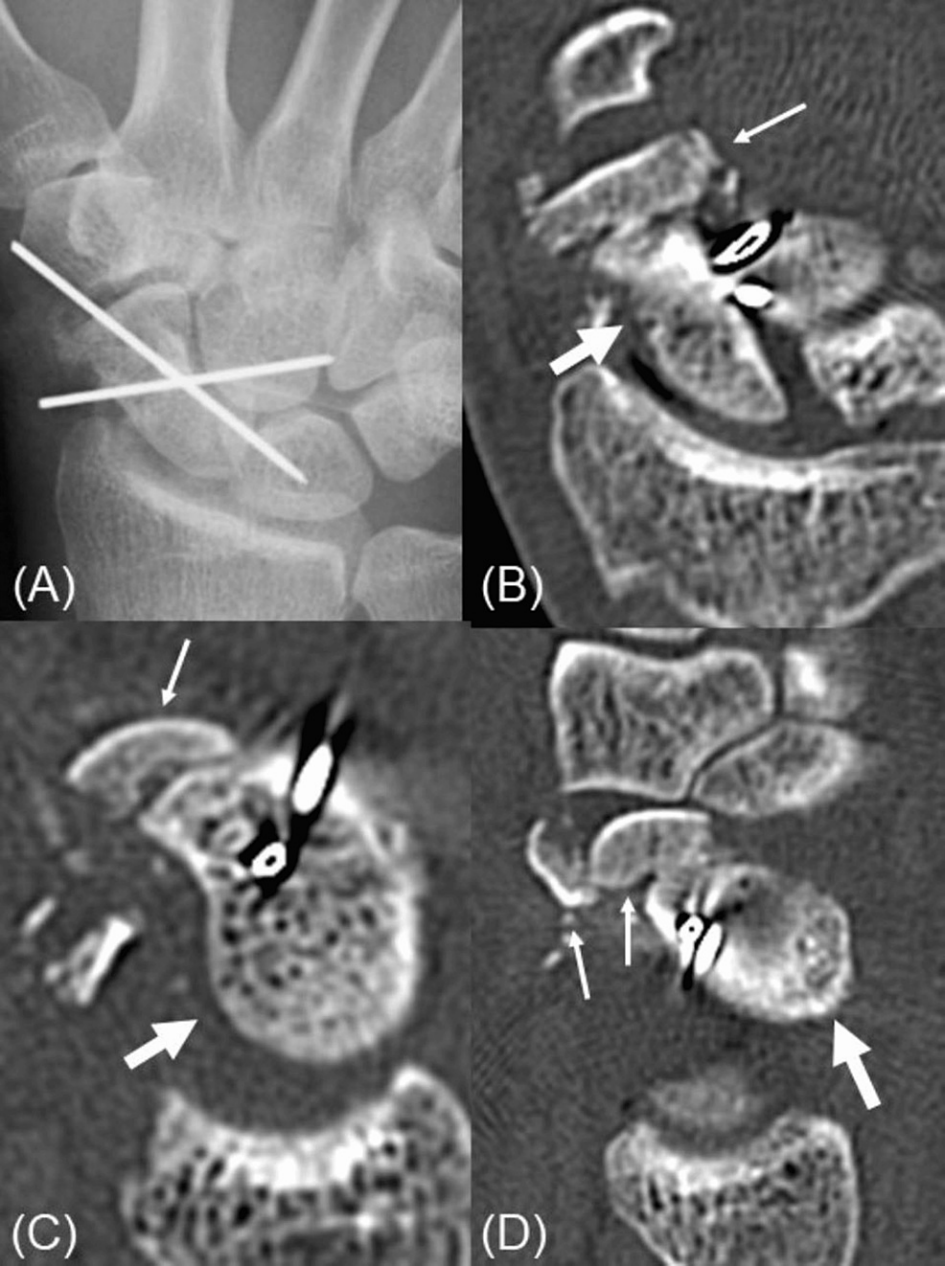
Radiographs and CT scans after initial treatment. Radiographs and CT scans after initial treatment display reduction and temporary fixation. The thick arrows show the proximal fragment of the scaphoid. The thin arrows show the distal fragment of the scaphoid. (A) Antero-posterior film of the left wrist shows the reduction of dislocation of the proximal fragment of the scaphoid and temporary fixation with Kirschner wires. (B-D) Coronal and sagittal CT images show that the dislocation of the proximal fragment of the scaphoid has been reduced, but the distal fragments are comminuted and thin.

The initial treatment at the previous hospital included reducing the proximal scaphoid fragment dislocation and temporary fixation with Kirschner wires (Figure 3).

**Figure 4:**
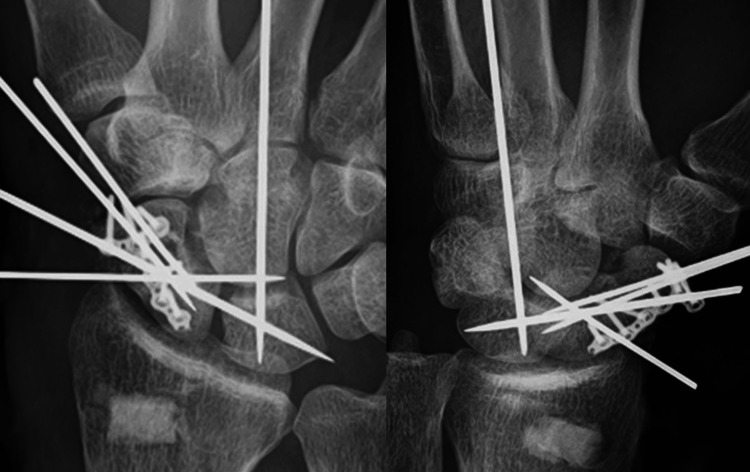
Postoperative radiographs. Postoperative radiographs showing fixation methods and grafting. We harvested autografts from the distal radius and filled the harvested area with artificial bone (beta-tricalcium phosphate).

However, due to the comminuted and thin distal fragments, instability remained. Eight days after the injury, surgery was performed under the brachial plexus block. A volar approach was used to access the scaphoid, and various fixation methods were applied. After we reduced the DISI (dorsal intercalated segment instability) deformity of the lunate, we temporarily fixed the lunate to the radius by a single Kirschner wire. Next, we reduced the dorsal dislocated proximal scaphoid fragment and fixed the proximal scaphoid fragment and the lunate with a single Kirschner wire. Since the body of the scaphoid was comminuted, the distal fragments were reduced using the trapezium as a template, and the distal and proximal fragments were fixed with two Kirschner wires. Then, bone defects were observed in the scaphoid body, so bone grafts from the distal radius were used to fill defects. A 1.5-mm T-shaped locking miniplate (Medartis AG, Basel, Switzerland) was placed on the volar surface to secure the autografts and stabilize the proximal and distal fragments (Figure 4).

**Figure 5:**
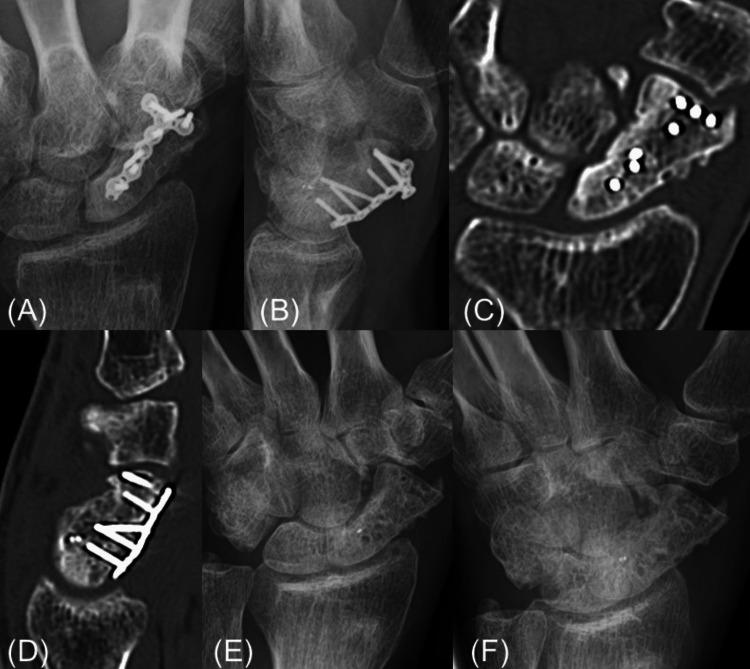
Radiographs and CT scans before and after locking plate removal. Radiographs and CT scans before and after locking plate removal, confirming union. (A, B) The radiographs show the scaphoid union. (C, D) The CT images similarly confirm the union. (E, F) The radiographs after the locking plate removal show the scaphoid union.

We used the T-shaped miniplate because the distal bone fragments were thin. Finally, the scaphoid-lunate and capitate-lunate were temporarily transfixed with Kirschner wires for relatively stabilizing the scaphoid (Figure 4). The scapholunate ligament was irreparable. The two Kirschner wires that temporarily fixed the main distal and proximal fragments were left in place and implanted subcutaneously. The open wound was covered with a rotational flap. Postoperatively, the wrist was immobilized with a volar splint for six weeks. The Kirschner wires temporarily fixing the capitate-lunate and scaphoid-lunate were then removed, and hand therapy was initiated. Four months postoperatively, the two subcutaneous Kirschner wires were removed. He returned to his previous work at seven months postoperatively. Although there were no symptoms of plate impingement, the locking plate was removed at 11 months postoperatively (Figure 5).

At a final follow-up 14 months after the operation, the range of motion (ROM) of the wrist was 70° extension and 55° flexion, and the grip strength was 81% of the uninjured side. The Mayo wrist score was 85 points. The categorical rating according to the Mayo wrist score was good (80 to 89 points).

## Discussion

Although comminuted scaphoid fractures or isolated scaphoid dislocations have been reported in a small number of cases [6,7], open comminuted scaphoid fracture dislocations are very rare and have not been reported in the literature. The mechanism of scaphoid dislocation is commonly hyperextension with axial loading of the wrist, but the injury, in this case, appears to have been caused by a direct blow (external force) to the scaphoid by the press machine. In cases like this, the treatment challenge arises from poor soft tissue conditions, comminuted fractures, and bone defects, making bone union difficult to achieve.

Treatment of scaphoid nonunion with locking plates has been described previously. Biomechanical studies have reported that locking plate fixation has superior anti-rotational forces than fixation with a headless compression screw [8] and equal or better resistance to axial pressure loading [9]. Although there have been previous reports that plate fixation did not have superior mechanical characteristics to headless compression screws [10], plate fixation seems to be the most consistent biomechanical form of osteosynthesis with a high level of fragment stability and safe bone graft fixation maintaining the scaphoid length [9].

As a treatment for this intractable injury, first, temporary wire fixation between carpal bones was performed for carpal interosseous ligament injuries due to dislocation, and one-stage free bone grafting was performed for bone defects. We performed bone grafting from the distal radius because of a previous report that bone grafting from the distal radius and from the iliac bone had similar fusion rates in the treatment of scaphoid nonunion [11], and because the procedure could be performed under brachial plexus blocks. Furthermore, to perform firm fixation while maintaining the scaphoid length, fixation with a locking plate was performed [9]. In addition, the scaphoid was indirectly fixed and rested by temporarily fixing the midcarpal joint. Fortunately, as a result, bone fusion was achieved. To avoid radiocarpal plate impingement and tendon rupture [12], we considered the possibility that the plate was limiting wrist flexion based on the report of impingement symptoms with wrist flexion of 60° or more [4]. Thus, we removed the locking plate without mechanical symptoms, but there was no improvement in the range of motion. Therefore, it may not have been necessary to remove the plate. In addition, considering the stability of the plate, leaving in place the Kirschner wires that temporarily fixed between the main fragments of the scaphoid may have been over-treatment. It is possible that the temporary wire fixation to the scaphoid and the midcarpal joint was not also necessary. Thus, we may have treated this patient with excessive fixation methods. Therefore, it seems that we should consider what would be a better internal fixation method for this injury. We believe that fixation with a plate, free bone grafting, and temporary fixation between the scaphoid and lunate were at least necessary.

## Conclusions

We experienced a rare case of an open comminuted scaphoid fracture dislocation. Such a case was not reported in the previous literature to our knowledge. This case was expected to have difficulty with bony fusion due to the poor soft tissue condition, comminuted fractures, and bone defects. Nevertheless, successful bone fusion was achieved with volar locking plate fixation combined with primary free bone grafting from the radius. Our case report may be a valuable resource for treatment of similar injuries.
